# The role of Peyer’s patches in synchronizing gut IgA responses

**DOI:** 10.3389/fimmu.2012.00329

**Published:** 2012-11-07

**Authors:** Nils Y. Lycke, Mats Bemark

**Affiliations:** Mucosal Immunobiology and Vaccines Center, Department of Microbiology and Immunology, Institute of Biomedicine, University of GothenburgGothenburg, Sweden

**Keywords:** gut IgA, cholera toxin, germinal center re-utilization, Peyer’s patches, B cells, germinal centers

## Abstract

Because Peyer’s patches (PP) are the main inductive sites for gut IgA responses we have focused this review on what we know about the function of PP germinal centers (GC). The vast majority of IgA gene sequences in the gut lamina propria (LP) are heavily mutated arguing for an origin in GC. Because PP GC formation is dependent on the presence of CD4 T cells, we speculate that all IgA responses in the normal gut are directly or indirectly T cell-dependent (TD). We hypothesize that the CD4 T cell involvement in gut IgA responses against the microbiota is different from that in systemic responses since cognate T–B cell interactions appear not to be required. In the absence of cognate interactions the function of CD4 follicular helper T cells (Tfh) in PP GC is unclear. However, production of IL-21 and IL-6 is more pronounced than in peripheral lymph nodes. Importantly, we discuss how multiple PP are involved in generating specific IgA responses to TD antigens given orally. Recently we found that oral immunization with NP-hapten conjugated to cholera toxin (NP-CT) stimulated a strong highly synchronized, oligoclonal and affinity matured IgA response. This was achieved through re-utilization of GC in multiple PP as GC IgA B cells emigrated into already established GC. Clonally related B cells were present in both inductive and effector lymphoid tissues in the gut and clonal trees involving multiple PP could be constructed in individual mice. Through adoptive transfer of B1-8^hi^ NP-specific B cells we demonstrated that GL7^+^ PP B cells could enter into pre-existing GC in PP, a process that was antigen-dependent but did not to require cognate Tfh interactions. Finally, we discuss the role of PP GC for the generation of memory B cells and long-lived plasma cells in the light of contrasting findings regarding IgA memory development to colonizing commensal bacteria versus that to oral immunization with enteropathogens or TD antigens.

## INTRODUCTION

**FIGURE 1:**
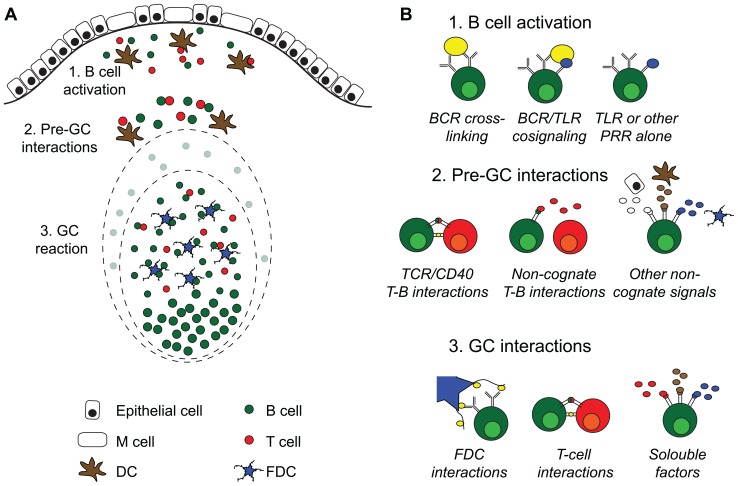
**Activation of B cells and germinal center formation in Peyer’s patches. (A)** Intestinal T cell-dependent antigens enter the gut via the oral route or are locally produced by the gut microbiota. Peyer’s patches (PP) are covered with a specialized follicle-associated epithelium hosting M cells that can transport antigen into the subepithelial dome. Here, B cells and DC encounter antigen, leading to B and T cell activation. After interactions with cells and soluble factors outside of the germinal centers (GC), B cells enter into preformed GC where they proliferate and expand before seeding the gut with IgA producing plasma cells. **(B)** Systemic T cell-dependent responses strictly rely on two signals, antigen cross-linking and TCR/CD40 mediated cognate interactions with antigen-specific T cells, before *de novo* GC formation take place. After that, the cells are dependent on direct interaction with follicular dendritic cells (FDC) and T cells in addition to soluble factors in the GC milieu for class switch recombination and somatic hypermutation followed by affinity maturation. GC are continuously present in PP, and antigen-specific B cells enter into preformed GC rather than to form new ones. PP GC can form in the absence of antigen-specific cross-linking, and although T cells are needed for their formation, the role of cognate interactions with T cells is unclear. This suggests that signals from pattern-recognition receptors (PRR), i.e., toll receptors (TLR), can substitute and/or collaborate with BCR cross-linking during B cell activation, and that non-cognate interactions with T cells and/or soluble factors may substitute for CD40 signals. Somatic hypermutation of antibody genes is a feature shared between systemic and PP GC. However, whereas antigen-specific B cells can affinity mature in both, we do not know if the cellular interactions that influence PP GC are the same as those governing GC at systemic sites.

To protect us against infections and to maintain homeostasis with the microbiota the gut relies on secreted IgA antibodies (Russel and Kilian, 2005). Given that the vast majority of B cells in the gut immune system are activated and undergo class-switch recombination (CSR) from IgM to IgA in the Peyer’s patch (PP) we need to expand our knowledge about the function of PP and especially its germinal centers (GC; Macpherson et al., 2008). Whereas, comparative studies in germ-free and wild-type mice have demonstrated that a large proportion of IgA plasma cells in the gut lamina propria (LP) are activated by antigens or molecular patterns associated with the microbiota, little is known as to whether these responses should be considered T cell-independent (TI) or T cell-dependent (TD; Fagarasan et al., 2010; Hapfelmeier et al., 2010; Bemark et al., 2012). For example, we have documented in CD40-deficient mice that, irrespective of a complete absence of T–B cell cognate interactions, these mice exhibit near normal levels of IgA plasma cells in their gut LP (Bergqvist et al., 2006). Hence, while lacking GC the CD40-deficient mice still promote IgA CSR in the PP, but gut IgA plasma cells in the LP have not undergone somatic hypermutation (SHM), in accordance with a TI differentiation pathway (Bergqvist et al., 2010). In contrast, gut LP IgA gene sequences revealed substantial SHM in wild-type mice, suggesting that they, indeed, had an origin in GC in the PP and appear to have been under the influence of CD4 T cells (Bergqvist et al., 2010; Bemark et al., 2012). Other recent studies in both humans and mice have also clearly documented that IgA gene sequences in general are highly mutated (Barone et al., 2011; Benckert et al., 2011; Lindner et al., 2012). This raises the question as to what the nature of the CD4 T cell influence could be (**Figure 1**).

Our findings in CD40-deficient mice argue that while cognate interactions are not required for B cells recognizing microbiota-associated TI-type antigens to undergo IgA CSR, the formation of GC is critical for SHM and affinity maturation. Thus, in this sense, these IgA B cell responses in the normal gut are dependent on GC and, therefore, indirectly dependent on CD4 T cells. However, this dependence is most likely not a cognate interaction. This interpretation finds support in a study by Casola et al. (2004) showing that B cells carrying antigen non-recognizing surrogate Ig receptors can establish GC in the PP, a process which was found to rely on non-cognate T cells and the presence of the microbiota. Thus, the microbiota could promote GC formation in PP independently of B cell antigen-recognition and CD4 T cells supported this process. This finding argues that tonic non-antigen mediated B cell receptor (BCR) signals are sufficient for GC formation in PP. Possibly, alternative activation pathways, such as TLR signaling, are sufficient to drive B cell activation and GC formation in PP (Casola et al., 2004). However, if this is a major pathway in wild-type immunoproficient mice, it would lead to a massive presence of polyclonal IgA plasma cells in the LP, a notion that is not supported by gene sequencing data. Rather this data indicate an accumulation of highly mutated oligonal IgA repertoires with increasing age (Lindner et al., 2012).

If cognate T–B cell interactions are not required, what is then the role of the CD4 T cell? An elegant study by Wei et al. (2011) demonstrated that failing to accumulate appropriate SHM was associated with dysfunctional IgA antibodies, which lead to bacterial overgrowth and translocation, resulting in hyperplasia of GC in PP and ILF. Hence, unmutated, presumably low affinity, IgA antibodies against the microbiota are less effective in protecting the host as the mucosal barrier is perturbed. Taken together, it, thus, appears that functional, anti-inflammatory gut IgA-responses against the microbiota are critically dependent on CD4 T cell influences and, therefore, are not archetypical TI-responses.

The fact that we normally associate GC with the development of long-lived plasma cells and memory B cells raises the question if memory development occurs against antigens associated with the microbiota. This notion was recently challenged by a study that demonstrated a lack of recall IgA responses against bacteria that had transiently colonized the gut intestine of germ-free mice (Hapfelmeier et al., 2010). Although long-lived plasma cells developed it appeared that IgA memory B cells could not develop against the bacterial antigens in these mice. Considering that the generation of long-lived plasma cells and memory B cells are the result of differentiation in GC and that the normal mouse PP continuously exhibit GC this observation is unexpected (Tangye and Tarlinton, 2009). We have observed a significant number of GC also in germ-free mice (Bergqvist et al., unpublished observation). But we do not know to which extent antigen-specific IgA B cells against the bacteria in the transiently colonized mice were generated in PP GC, albeit GL7^+^ B cells were observed (Hapfelmeier et al., 2010). Conversely, ample documentation shows that oral immunization with TD-antigens generate IgA memory B cells. For example, oral immunization in mice with cholera toxin (CT) effectively stimulates long-lived plasma cells in the LP and memory B cells that protect against CT-challenge even 1.5 years later (Lycke and Holmgren, 1987). Likewise, studies on gut immunity to rotavirus have clearly documented priming of a specific IgA memory B cell response, which protected against rotavirus challenge through a mechanism that appeared to depend on the frequency of rotavirus-specific memory B cells in the GALT (Moser and Offit, 2001). Thus, evidence both in support of and opposing gut IgA B cell memory development has been published. If GC and CD4 T cells indeed are involved in responses against the microbiota then a natural question must be how is it possible for the GC reaction to differentially support memory B cell development against TD-antigens but not to antigens belonging to the microbiota?

The present review will address these fundamental aspects of PP function and discuss recent findings that help shed light on the questions raised in the introduction. It is an attempt to discuss the role of GC in PP in the light of what we currently know about mutations in antigen-specific gut IgA responses or based on observations using IgV_H_-chain gene sequencing of gut IgA. Special focus will be given to how synchronization of IgA B cell responses in the GALT may occur, as we have recently published a study using the well characterized hapten (4-hydroxy-3-nitrophenyl)acetyl(NP) conjugated to CT for studies aimed at dissecting the different processes that critically are needed for a strong synchronized gut IgA response (Bergqvist et al., 2012).

## GERMINAL CENTERS AND THEIR ROLE IN ANTIGEN-SPECIFIC RESPONSES

The formation of GC during immune responses plays a crucial role for the development of class-switched antibodies and the formation of B cell memory (Tangye and Tarlinton, 2009). Following antigen-recognition, B cells in the follicle will form GC together with follicular dendritic cells (FDC), which are key elements in the reaction (Schwickert et al., 2007). As the GC reaction progresses SHM of the BCR randomly generates relatively higher affinity variants from which plasma cells and memory B cells are selected (Berek et al., 1991). B cells expand in the dark zone and later undergo positive selection in the light zone, interacting with antigen presented as immune complexes on the FDC (Allen et al., 2007; Victora et al., 2010). Humans as well as mice lacking CD40–CD40L interactions fail to develop GC and characteristically present with hyper-IgM syndrome, i.e., high levels of IgM but little switched antibodies in serum, no TD antigen responses and lack of switched memory B cells (Kawabe et al., 1994; Xu et al., 1994; Weller et al., 2001). CD40 and CD40L interactions occur at the border between the B and T cell areas, where these cells meet after antigen-specific activation (Garside et al., 1998). Importantly, this interaction not only seems to be required for GC formation but also promotes GC maintenance, as blockade of CD40 disrupted existing GC (Han et al., 1995).

The constitutive GC formation that is observed in PP and tonsils, and to a lesser extent, in mesenteric lymph nodes (MLN) may be driven through signals other than those critically important for GC reactions in systemic lymphoid tissues. We have demonstrated that no GC develop in PP of CD40-deficient mice, but how BCR signaling or other receptor interactions critically influence PP GC formation remains to be further investigated (**Figure 1**). As aforementioned, GC in PP have been observed independently of BCR-recognition and hence the role of TLR signals may be critical in PP for GC development and for maintaining normal homeostatic function of the gut IgA immune system (Casola et al., 2004). To what extent such BCR-independent induction of gut IgA is the source for “natural IgA” that has been claimed to be part of the gut mucosal barrier function is still poorly understood (Kramer and Cebra, 1995; Macpherson et al., 2000). Recent progress in IgA gene sequence analysis using next generation sequencing techniques will most probably help answer some of the confusions around the concept of low-affinity binding “natural IgA,” encoded by poorly mutated near germ-line sequences (Lindner et al., 2012).

## PP IS THE MAIN IgA B CELL INDUCTIVE SITE IN THE GALT

Whereas most investigators agree that PP are the main inductive sites for gut IgA responses, irrespective of if they are elicited by antigens that belong to the microbiota or more classical TD antigens, a particular problem with the current view on the function of PP is how we can explain their role in driving protective gut LP IgA responses. IgA antibodies must be of sufficient quality to effectively protect against, e.g., pathogens or toxins (Russel and Kilian, 2005). Depending on variability between mouse strains, the average small intestine has between 8 and 12 PP preferentially located to the proximal and distal parts of the small intestine (Macpherson et al., 2008). In addition, the colon has patches that contain large aggregates of lymphocytes that in many respects appear to mimic the PP in their function (Dohi et al., 1999; Bergqvist et al., 2010). Antigen-activated PP B cells leave the tissue and migrate via the draining lymph to the MLN and then continue via ductus thoracicus to the blood from where they home back to the intestine (Macpherson et al., 2008). A consequence of this model is that in mice 10–12 PP would actively be delivering B cell blasts and plasma cells to the gut LP, and that these would represent diverse unique B cell clones with varying affinities. The protective efficacy of gut IgA, therefore, could vary greatly along the gut intestine, and in humans that often have more than 200 PP, this notion would be even more difficult to reconcile with an effective protective IgA response (Cornes, 1965). However, if we instead predict that the system is synchronized between PP and that only high affinity IgA cells are selected to dominate in the gut LP, it is easier to explain how gut IgA antibodies can effectively protect. One possibility could be that synchronization occurs in the MLN, but a recent study failed to ascribe an essential role for MLN in developing strong gut antitoxin IgA responses (Hahn et al., 2010). Thus, presently we largely lack convincing data to explain if and how synchronization of the gut LP IgA response can be achieved. Therefore, we recently directly addressed this question in a study where we focused on gut antigen-specific IgA B cell responses to the NP-hapten after oral immunization with NP-CT, a TD antigen (Bergqvist et al., 2012).

## MULTIPLE PP GERMINAL CENTERS SYNCHRONIZE AND EXERT QUALITY CONTROL OF GUT IgA ANTIBODY RESPONSES

**FIGURE 2:**
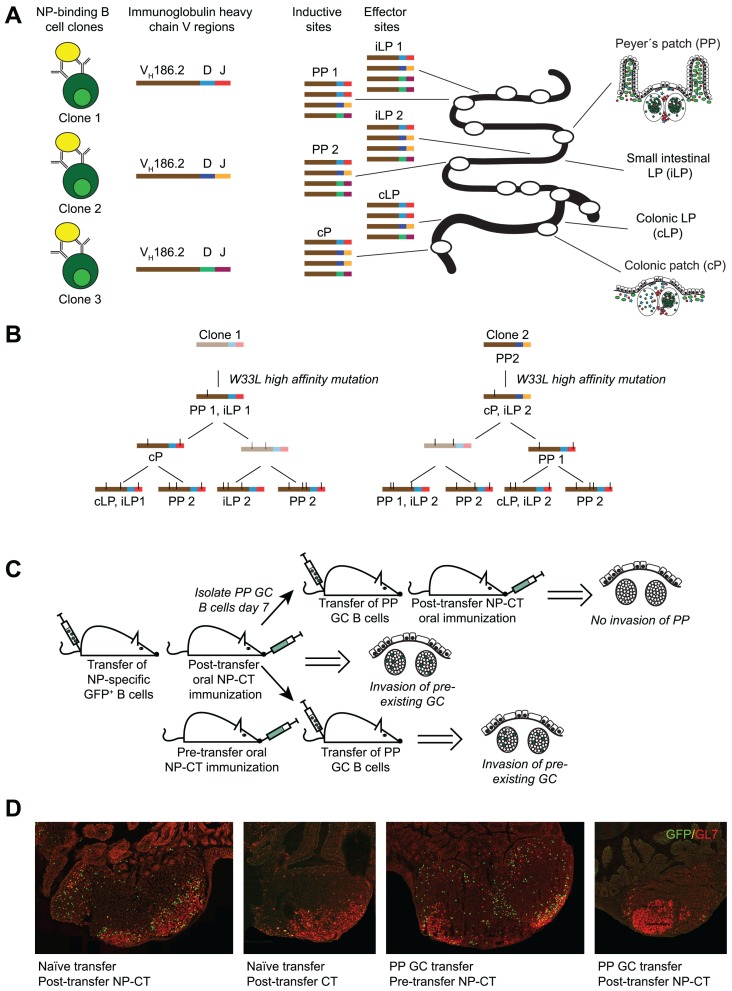
**Antigen-dependent invasion of preexisting Peyer’s patch (PP) germinal centers as a mechanism to synchronize gut responses.** We recently published a study that demonstrated that IgA responses are synchronized through invasion of pre-existing germinal centers in PP (Bergqvist et al., 2012). This conclusion was based on three observations. **(A)** The response to NP was dominated by B cells carrying a specific heavy chain V region (*V*_H_*186.2*). Because antibody sequences differ in the CDR3 region it allows for clonal analysis of the response. Sequencing of *V*_H_*186.2-IgA* genes from wild-type mice orally immunized with NP-CT demonstrated that the response to NP was highly oligoclonal and that B cells carrying identical CDR3 regions were simultaneously present at distinct inductive sites. This suggested that activated B cells from one inductive site, i.e., one PP GC, could distribute to multiple PP as well as seed the lamina propria (LP). **(B)** When clonal trees were constructed, it was evident that one specific mutation (W33L), which is known to increase the affinity 10-fold, appeared early in the response it also was represented at other inductive sites. This way the IgA response was effectively synchronized. **(C,D)** To confirm that PP GC could be invaded by GC B cells, a transfer experiment was conducted using GFP-expressing NP-specific B cells. We found that when naїve splenic B2 cells were transferred into wild-type recipients that were subsequently orally immunized with NP-CT, GFP^+^ cells invaded preexisting PP GC. By contrast PP GC B cells could not invade other PP after adoptive transfer into syngeneic naїve recipient mice unless oral NP-CT was given 24 h prior to cell transfer. Thus, antigen, but not T cell help, appeared to be required for migration of PP GC B cells into already existing heterologous GC.

The main finding of our study was the observation that extensive clonal lineage trees of NP-specific IgA B cells and plasma cells could be identified both at inductive and effector sites of the gut immune system following oral immunization (Bergqvist et al., 2012). In particular, when analyzing NP-specific IgA sequences that used the *V*_H_*186.2* V region, we observed that clonally related B cells were found not only within single PP and the gut LP, but that the same clone was present in multiple PP in the same mouse, suggesting that the expansion of these clones was synchronized and a consequence of an antigen-dependent selection process (**Figure 2**). Repeated oral immunizations resulted in enhanced antibody affinity and we could follow the acquisition of a particular mutation in the CDR1 region, resulting in a 10-fold enhanced affinity of anti-NP IgA antibodies, with increased number of oral immunizations (Bergqvist et al., 2012). Remarkably, the frequency of anti-NP IgA cells with the affinity-enhancing *V*_H_*186.2* W33 to L33 mutation in the CDR1 region increased more rapidly in the PP than in the gut LP. After the second dose of oral NP-CT only 20% of gut LP IgA carried the mutation compared to 60% in PP, while a third oral immunization resulted in that 60% of the NP-specific IgA cell clones exhibiting high affinity maturation also in the LP. Thus, the gut immune system effectively selected for higher affinity with repeated oral immunizations, and a small number of anti-NP IgA clones dominated the response after three oral immunizations. In addition, we observed that clonally related IgA cells were distributed to the LP of both the small and large intestine, albeit the frequency of related clones was higher in the small intestine than between the small and large intestine, suggesting that there was some compartmentalization of the IgA response. This notion finds support in the work by Lindner et al. (2012), where clones distinctively clustered separately to the small or large intestine.

The extensive lineage trees and the oligoclonal domination of anti-NP IgA cells after repeated oral immunizations indicated that the gut IgA response was not only strongly regulated but also synchronized between PP. This synchronization must be achieved through simultaneous antigen-driven selection in multiple PP, which suggested that antigen-activated NP-specific IgA B cells from one PP were distributed to GC of multiple PP. Hence, we hypothesized that following the priming immunization, GL7^+^ NP-specific B cells could leave the GC in one PP and migrate to already established GC in other PP. This was indeed supported by experiments that followed the distribution of NP-specific B1-8^hi^ GFP B cells in an adoptive transfer model, which demonstrated that NP-specific B cells first expanded in proximal PPs after a priming immunization while after a third oral immunization these cells were equally frequent also in distal PP. Furthermore, when we transferred GL7^+^ B cells isolated from PP after one oral priming immunization, these could migrate into GC in multiple PP of the recipient mouse provided NP-CT had been administered prior to the transfer (**Figure 2**). Thus, B cells from GC of one PP can migrate into already established GC in multiple PP – attesting to the notion that synchronization of the gut IgA response occurs through reutilization of already established GC in multiple PP. These data for the first time provide evidence that there is synchronization of gut IgA responses, and that these responses are dependent on the repeated exposure to antigen and effective distribution of antigen-activated PP B cells into multiple PP.

## REQUIREMENTS FOR REUTILIZATION OF GERMINAL CENTERS IN PP

These findings pose a number of questions as to what is required for the PP synchronization system to operate? Firstly, we can conclude that the migration of NP-specific B cells into already established GC required the presence of antigen, as no invasion of NP-specific B cells was observed in mice orally immunized with CT only (Bergqvist et al., 2012). The finding that newly activated B cells can reutilize already existing GC has previously been elegantly shown by Schwickert et al. (2009) and excellently discussed by Or-Guil et al. (2007). Consistent with our results, Schwickert et al. (2009) demonstrated that sequential administration of two different antigens lead to efficient invasion of existing GC in draining lymph nodes by heterologous high affinity B cells.

However, a prerequisite for an effective reutilization system in the gut is that early GC emigrants, and not only newly activated B cells, can invade already established GC in PP (Tarlinton, 2006; Or-Guil et al., 2007). To test the ability of activated B cells to do this, we transferred GL7^+^ PP GC B cells 7–9 days after an oral priming immunization (Bergqvist et al., 2012). In this case, antigen was still required for invasion but the presence of CD4 T cell help appeared not to be critical as antigen administration only 24 h prior to transfer of GL7^+^ B1-8^hi^ GFP B cells was sufficient, a time frame that excludes the generation of effective CD4 T cell help. Thus, for localization of GC-expanded GL7^+^ PP B cells into already existing heterologous GC in multiple other PP presence of antigen but not cognate interactions with CD4 T cells was essential. This contrasted to the previous studies by Schwickert et al. (2009) that indicated that the reutilization required that B cell epitopes/antigens shared the same carrier protein, suggesting that cognate interaction with shared CD4 T cell help was critical. Hence, GL7^+^ B cells have different requirements than newly activated B cells, or, alternatively, PP GC are unique and different from GC in lymph nodes and the spleen with regard to CD4 T cell involvement. Reutilization of PP GC could, for example, be dependent on non-cognate functions of CD4 T cells as found with the localization of LMP2A-expressing B cells to PP GC (Casola et al., 2004). One important factor that could influence this pattern is IL-21 (Pistoia and Cocco, 2009). Whereas the formation of GC requires CD40 signals in both PP and other secondary lymphoid tissues, T cell subsets distinct from archetypical CD4^+^ T cells appear to be present in PP. For example, Seo et al. (2009) demonstrated that IL-21 was more strongly expressed by CD4 T cells in PP than in spleen and that in the context of TGFβ the dominant isotype produced was IgA, whereas IgG2b antibody production was effectively suppressed by IL-21. Whether other aspects than IgA CSR were affected by IL-21 in PP was not further investigated in this study, but Dullaers et al. (2009) found that recombinant TGFβ and IL-21 can drive naїve human B cells toward a mucosal phenotype. It is therefore plausible that IL-21 in PP promotes survival, proliferation, and differentiation of activated B cells in PP GC.

Follicular helper T cells (Tfh) in the PP have not been studied in detail and presently we do not know their role in supporting B cells that reutilize GC and that recognize heterologous antigens (Hashiguchi et al., 2011; Danisch et al., 2012). Clearly, PD-1 is critical for the generation of Tfh in the PP, and PD-1-deficient mice have a reduced ability to develop gut IgA antibodies that recognize the microbiota, resulting in reduced bacterial binding capacity and breaching of the mucosal barrier (Kawamoto et al., 2012). This observation identifies that Tfh in the PP GC are critically needed for functional IgA production, and argues in favor of that also IgA B cells recognizing TI-type of gut antigens, belonging to the microbiota, require CD4 T cell help to acquire SHM and produce protective antibodies. Diverse T cells are present in GALT, including T_H_1, T_H_2, T_H_9, T_H_17, T_reg_, Tr1, T_FH_, and CD8^+^ α/β T cells as well as γ/δ cells, and there is considerable functional overlap between these T cells with regard to their ability to support B cell differentiation (Hayday and Gibbons, 2008; Wan, 2010). However, this process may not require cognate interactions, but may rather rely on IL-21 and other factors produced by PP Tfh. Recent data suggest that the origin for Tfh cells in the GALT may be unique in that they could be derived from FoxP3-expressing T_reg_ cells, rather than from T_H_1 or T_H_2 cells, although both these latter subsets have been found to function as Tfh in PP (Garside et al., 2004; Tsuji et al., 2009). An intriguing possibility may be that activated B cells, migrating into already established PP GC, do not require cognate T cell help, but rather interact with CD40L-expressing PP Tfh in the absence of cognate interactions. This could be a unique function of PP Tfh – to provide contact-dependent CD40L/CD40 signals but not to engage into TCR/MHC interactions. In this case, migrating activated PP B cells would require BCR-recognition and CD40L-expressing Tfh to proliferate in already established PP GC. What the consequences of such CD40- and BCR-driven B cell expansion and differentiation could be, as opposed to a cognate-dependent process in the spleen or lymph nodes, can only be a matter of speculation. Perhaps, the large number SHM in gut IgA cells bear witness of a less stringently regulated system in PP GC where B cells will continue to contract mutations even after high-affinity binding is achieved. Nevertheless, future studies will address this important question and hopefully we will better understand how an ensemble of GC in multiple PP rather than individual GC, critically functions to synchronize protective gut IgA responses and what role Tfh cells have to support the development of functional IgA antibody production when cognate T–B cell interactions are not required.

## IgA MEMORY B CELL DEVELOPMENT FOLLOWING ORAL IMMUNIZATION IN THE ABSENCE OR PRESENCE OF GERMINAL CENTERS

The ability of the gut immune system to protect the mucosa against pathogens by long-term production of antigen-specific IgA antibodies is pivotal to mucosal vaccine development. A recent study by Macpherson and colleagues addressed the importance of mucosal memory after mono-colonization of germ-free mice with a commensal bacterial strain dependent on nutrients not present in the mammalian hosts (Hapfelmeier et al., 2010). Using this reversible colonization model, the authors found that repeated bacterial exposures gave rise to increasing specific SIgA titers in an additive rather than a synergistic manner, failing to exhibit a classical prime-boost effect. Interestingly, in the absence of competing antigens, the half-life of the LP plasma cells was very long, whereas in the presence of other bacteria that triggered the formation of new IgA plasma cells, the lifespan of the IgA plasma cells in the gut LP was dramatically reduced (Hapfelmeier et al., 2010). Based on these observations, the authors concluded that memory B cells did not develop, and that, although LP plasma cells had the potential for longevity, they were short-lived in conventionally reared mice.

These conclusions may be relevant for gut B cell responses driven by the microbiota, but they contrast with findings after oral immunizations with TD-antigens, as discussed below. Because GC and SHM play a critical role for both types of IgA responses we believe it will be important to understand if there are fundamentally different regulatory principles that operate in the PP GC depending on the type of antigen that drive the B cell response (Bemark et al., 2012). Whereas memory B cells can form independently of GC, such memory B cells, mostly recognizing TI-antigens, are of a different quality and harbor less mutations and affinity maturation than GC-dependent memory responses (Toyama et al., 2002). To what extent specific anti-bacterial gut IgA responses are more or less mutated still need further investigation, but recent data suggest that they are more mutated and carry GC-dependent features (Lindner et al., 2012). Hence, to understand why commensal bacteria do not stimulate memory B cell development, but TD-antigens, such as CT, do, we will have to predict that memory B cell development in PP is not an intrinsic property of the GC, but rather a quality that is under additional regulatory control. In this context, interactions with Tfh cells may be critical.

Tfh cells have been shown to induce a memory B cell phenotype in GC B cells cultured *in vitro* and can produce IL-21, IL-10, IL-6, and IL-4, which all have been implicated as instructive signals in GC plasma cell and memory B cell differentiation (Casamayor-Palleja et al., 1996; Hashiguchi et al., 2011; Shlomchik and Weisel, 2012). Given that PP Tfh appear to be different from archetypical Tfh in systemic secondary lymphoid tissues, we propose that they may provide instructive signals to B cell memory development even in the absence of cognate interactions. Hence, failure to develop memory B cells, concomitant with a strong induction of long-lived plasma cells, as reported by Hapfelmeier et al. (2010) may be a consequence of what type of, or lack of, Tfh activity that the commensal bacteria induced. In conventionally reared mice this situation may not have occurred as a multitude of commensal species are present in the intestine and potentially can stimulate a broadly functional Tfh population in PP GC. Of note, the composition of the microbiota plays an important role in shaping gut T cell functions as evident in studies showing, for example, that segmented filamentous bacteria stimulate the development of T_H_17 cells, which dramatically could influence activation of mucosal B cells (Ivanov et al., 2009; Datta et al., 2010). In addition, we must consider that transiently colonizing germ-free mice with *E. coli* K-12 bacteria most probably will lead to a TLR-mediated hyper-responsive state, which could facilitate BCR signaling and result in a TI-type of response, lacking memory development. Nevertheless, future studies are much needed to resolve this important and puzzling question.

Humans indeed maintain a sizable proportion of blood B cells that appear to be circulating IgA^+^ memory cells (Klein et al., 1998; Harris et al., 2009; Tengvall et al., 2010). Furthermore, IgA-producing plasma cells reactive to rotaviral antigens were isolated from the duodenum of 9 out of 10 human adults that had not recently experienced rotavirus, arguing for the presence of long-lived plasma cells in the gut LP (Di Niro et al., 2010). In mice, we know from previous studies that anti-toxin IgA plasma cells can reside in the gut LP more than 6 months after oral immunizations with CT (Lycke and Holmgren, 1987). However, after 12 months specific plasma cells had largely disappeared, but memory B cells in the GALT could easily be triggered by a challenge-immunization with oral CT, giving rise to a vigorous anti-toxin IgA plasma cell response in the gut LP within 3 days upon re-challenge (Lycke and Holmgren, 1987). The duration of gut anti-toxin protection, thus, clearly reflected the ability of long-lived IgA memory B cells to elicit a rapid recall response to a renewed exposure to the antigen.

We have previously demonstrated that memory B cells from the GALT can be adoptively transferred to naїve syngeneic recipient mice, and upon an oral immunization with CT, elicit a strong gut IgA response in the LP (Lycke and Holmgren, 1989). More recently we have observed that these transferable memory B cells are present exclusively among CD80^+^ B cells from spleen, MLN, and PP even 1 year after oral priming immunizations (Andersson et al., 2007; Bemark et al., 2011; Bemark et al., unpublished observation). Others have demonstrated that adoptively transferred rotavirus-specific memory B cells can effectively clear the infection from rotavirus-infected RAG-deficient mice (Williams et al., 1998). Interestingly, in the latter study splenic memory B cells with the ability to clear rotavirus infection through SIgA production could be identified on the basis α_4_β_7_ integrin expression, whereas B cells lacking this integrin did not clear the infection. Taken together, it is clear that mucosal IgA responses against TD-antigens following infection or oral vaccination also effectively stimulate the development of antigen-specific memory B cells and long-lived plasma cells.

The generation of memory B cells after oral immunization raises several questions with regard to if, and in that case, how they differ from memory cells that are generated through systemic immunization. This question is not only important from a mechanistic point of view, but may also have important implications for the development of more effective mucosal vaccines. Moreover, if, as discussed above, B cells recognizing antigens from the microbiota enter PP GC, will they give rise to memory B cells and long-lived plasma cells, or are cognate B–T cell interactions needed? Given that the mucosal immune system holds several unique traits compared to the systemic immune system we would argue that it could also apply to the ability to support memory B cell and long-lived plasma cell development. There are indeed data to suggest that oral immunization can be more efficient than systemic immunization for the generation of long-term immune protection, especially against enteropathogenic infections (Lycke, 2012). Whether this is because of the expression of α_4_β_7_ integrin on mucosal memory B cells, as suggested by the rotavirus studies, or could be a function of other properties of these memory B cells compared to systemic memory B cells is poorly understood. Moreover, we know that oral immunization stimulate mucosal and systemic memory B cell development, whereas systemic immunization fails to stimulate mucosal memory responses (Lycke, 2012; Bemark et al., unpublished observation). Understanding what the difference is between the immunization routes is critical for vaccine development, in general, and for mucosal vaccine development, in particular. Presently, we lack information about the relationship between systemic memory IgG and mucosal memory IgA cells following oral immunization. Are these memory B cells clonally related and do they emanate from the same or different inductive sites? We are currently addressing these pressing questions using the NP-CT system and the B1-8^hi^ NP-specific IgH knock-in B cell adoptive transfer model described above, with the hope to defining which Tfh functions and molecular pathways that are involved in memory B cell development in the PP GC.

## CONCLUSION

The GC in the PP appears to be a unique site for expanding and synchronizing gut IgA B cell responses. We have found that clonally related antigen-specific IgA cells are distributed to both inductive (PP) and effector sites (LP) following oral immunization with a TD antigen, NP-CT. This was achieved through reutilization of already established heterologous GC in multiple PP for the expansion and selection of antigen-primed GL7^+^ B cells. In adoptive transfer experiments we observed that presence of antigen was critical for the migration of both newly activated and GL7^+^ B cells into the GC, while cognate Tfh interactions appeared not to be important, at least in the latter case. This raises the question as to the role of Tfh in PP GC. Recent studies in Irf4-deficient mice have clearly demonstrated that Tfh activity is required for GC formation in PP (Bollig et al., 2012). Hence, Tfh cells are clearly involved, but other studies suggest that non-cognate signals may be more important than cognate T–B cell interactions to support IgA B cell differentiation. Because most IgA gene sequences in the gut LP are heavily mutated it is thought that both TD and TI-antigen triggered IgA B cells have passed through PP GC. For example, IgA responses against the microbiota in CD40-deficient mice exhibit few mutations, but in wild-type mice it can be assumed that microbiota-reactive B cells have passed through GC as almost all IgA-producing plasma cells have undergone significant SHM, arguing in favor of a Tfh activity independent of cognate recognition in PP GC. Given that a GC origin appears to be shared by B cells responding to TD and microbial antigens, it would be expected that both types of responses are able to generate long-lived plasma cells and memory B cells. However, a recent study has provided evidence for a failure to develop B cell memory even after repeated transient bacterial colonization in germ-free mice. These conflicting findings point to that PP GC exhibit special conditions that are unique and completely different from those in GC in systemic peripheral lymph nodes. Future detailed studies will hopefully shed light on these questions and help resolve the confusing picture that currently is an impediment for a rational design of effective oral vaccines.

## Conflict of Interest Statement

The authors declare that the research was conducted in the absence of any commercial or financial relationships that could be construed as a potential conflict of interest.
